# Modeling the Soil Water Retention Curves of Soil-Gravel Mixtures with Regression Method on the Loess Plateau of China

**DOI:** 10.1371/journal.pone.0059475

**Published:** 2013-03-15

**Authors:** Huifang Wang, Bo Xiao, Mingyu Wang, Ming'an Shao

**Affiliations:** 1 Beijing Research & Development Center for Grass and Environment, Beijing Academy of Agriculture and Forestry Sciences, Beijing, China; 2 Center for Water System Security, Graduate University of Chinese Academy of Sciences, Beijing, China; 3 State Key Laboratory of Soil Erosion and Dryland Farming on the Loess Plateau, Institute of Soil and Water Conservation, Chinese Academy of Sciences, Yangling, Shaanxi, China; Lakehead University, Canada

## Abstract

Soil water retention parameters are critical to quantify flow and solute transport in vadose zone, while the presence of rock fragments remarkably increases their variability. Therefore a novel method for determining water retention parameters of soil-gravel mixtures is required. The procedure to generate such a model is based firstly on the determination of the quantitative relationship between the content of rock fragments and the effective saturation of soil-gravel mixtures, and then on the integration of this relationship with former analytical equations of water retention curves (WRCs). In order to find such relationships, laboratory experiments were conducted to determine WRCs of soil-gravel mixtures obtained with a clay loam soil mixed with shale clasts or pebbles in three size groups with various gravel contents. Data showed that the effective saturation of the soil-gravel mixtures with the same kind of gravels within one size group had a linear relation with gravel contents, and had a power relation with the bulk density of samples at any pressure head. Revised formulas for water retention properties of the soil-gravel mixtures are proposed to establish the water retention curved surface models of the power-linear functions and power functions. The analysis of the parameters obtained by regression and validation of the empirical models showed that they were acceptable by using either the measured data of separate gravel size group or those of all the three gravel size groups having a large size range. Furthermore, the regression parameters of the curved surfaces for the soil-gravel mixtures with a large range of gravel content could be determined from the water retention data of the soil-gravel mixtures with two representative gravel contents or bulk densities. Such revised water retention models are potentially applicable in regional or large scale field investigations of significantly heterogeneous media, where various gravel sizes and different gravel contents are present.

## Introduction

A large proportion of soils containing rock fragments are present in the world due to soil evolution and erosion [1], [2]. The highly variable gravel content or size in soilscape greatly increases the variability of the soil properties [3]–[5]. Knowledge of soil water retention curves (WRCs) is a prerequisite for modeling the fluxes of water and solutes in the vadose zone and consequently it is necessary to determine their spatial variability [6]–[8]. Since direct field measurements of WRCs are time-consuming and expensive, laboratory measurements continue to be the most frequent means of characterizing the vadose zone [9], [10]. Soil water retention data are typically obtained in laboratory for fine soils (<2 mm) using pressure cells, pressure-plate extractors, and centrifuge methods [11]–[13]. Several reports noted that water held by gravel cannot be neglected in the determination of water retention properties due to the significant porosity of gravel and the changed pore-size distribution [14], [15]. For example, the ironstone gravel contained a large amount of available water ranging from 0.03 cm^3^ cm^−3^ to 0.15 cm^3^ cm^−3^ [16], while the sandstone fragments and shale fragments held 0.11 cm^3^ cm^−3^ and 0.23 cm^3^ cm^−3^ available water, respectively [17]. For soil-glass mixtures, the volume of coarse lacunar pore increased with glass content when glass content was less than 50% [18]. As a large number of water retention curves for fine soils have been obtained in laboratory, gravel corrections for moisture retention in soil-gravel mixtures have been developed on the basis of WRCs of fine soils. For example, Gardner [19] used mass-based approach while Bouwer and Rice [20] used volume-based approach to make gravel corrections for water retention of soil-gravel mixtures [20]–[22]. Correction procedures are available to determine water retention properties for soil-gravel mixtures, but they have limited utility for soil-gravel mixtures with weathered gravels especially on high-suction range [23]. Pedotransfer functions for predicting WRCs have been developed from more easily measurable and more readily available soil properties [24]–[27]. Scheinost et al. [6] predicted WRCs for soils with a wide range of particles which included 2–67 mm gravel with a new pedotransfer function including more textural fractions, but this did not greatly improve the prediction precision [25], [28].

In order to quantify the effects of content and size of rock fragments on soil water retention and finally develop a new method for determining the WRCs of soil-gravel mixtures, especially for mixtures with weathered gravels and for WRCs at high-suction range, we investigated WRCs of a loess soil and gravels mixtures with various gravel contents and gravel sizes. The following constraints were soon evident in developing this new method: (1) To extend the practical use of the revised model, the water retention data of soil-gravel mixtures with weathered gravel should be obtained and the range of water potential should be extended to include very low values (high-suction). (2) To develop revised water retention models, the effects of gravel contents and gravel size on the effective degree of saturation (*Se*) of soil-gravel mixtures should be analyzed based on the measured WRCs data. The relationships between *Se* and gravel contents or bulk density of soil-gravel mixtures should be combined with a closed-form analytical equation such as the model of Brooks and Corey (BC-function) [29] or the van Genuchten equation (VG-function) [30]. (3) To validate the effectiveness of revised water retention models and simplify the procedure of parameter-obtaining, the way that the gravel size affects the shape parameters of revised models should be analyzed, and the shape parameters of the revised models should be obtained from the representative WRCs data of soil-gravel mixtures, such as that with two extremes values of gravel content range. Those parameters and the method of parameter-obtaining may give references for determining WRCs of other soil-gravel mixtures.

## Materials and Methods

### Ethics statement

No specific permissions were required for these sampling activities because the location is not privately-owned or protected in any way and the field activities did not involve endangered or protected species.

The samples were excavated from the soil profiles in 30 cm depth of Yaoxianliang in Tongchuan (108.93 E, 35.28 N, at 1570 m altitude) and Weihe river bank in Yangling (108.10 E, 34.25 N, at 437 m altitude), Shaanxi province, China. The soil in Yaoxianliang is recognized as Aric Regosol (FAO). The rock fragments in these soils are schists and shales (S) with brownish green in color and sheet-like shape. The other type of rock fragments sampled in Weihe river bank was pebble (P) from alluvial sediment. The rock fragments in sizes ranging from 2 to 10 mm were sieved and washed to remove the soil particles from their surface, and then sieved into three different diameter classes: 2–3 mm, 3–5 mm, and 5–10 mm. The weathering degree of the rock fragments was defined by observing their weathering characteristics in the field and observing the surface fissures and coarseness under a magnifier in the laboratory according to the Geotechnical Engineering Handbook [31]. The gravel weathering degree is described in Table 1.

**Table 1:** Mineral composition of soil and rock fragments sampled in this study.

Fine soil (<2 mm)				Rock fragments (2–10 mm)			
Texture	Grain (μm) content in volume (%)			Petrology	Mean density (g cm^−3^)	Shape	Weathering degree
Clay loam soil	0.02–2	2–20	20–2000	Shale clasts (S)	2.09±0.04	Flake, block	High
	17.9	39.4	42.7	Pebbles (P)	2.49±0.15	Sphere, ellipsoid	Low

The texture of the air-dried fine soil (<2 mm), from which the rock fragments (> 2 mm) and plant residues were removed, was measured by the Laser Diffraction Particle Size Analyzer (Mastersizer 2000, Malvern Instruments Ltd. in England). According to the International Soil Classification System, the disturbed soil sample was a clay loam soil (labeled with CL). The mean density of the rock fragments (*ρ_r_*) in 2–10 mm, determined using dividing masses by the corresponding volume of water, ranged from 2.09 to 2.49 g cm^−3^ (Table 1). It should be noted that the rock fragments were saturated in water before determining the water volume substituted by the rock fragments in a container with scale when measuring *ρ_r_*. The saturated water contents (*θ_s_*) of the gravels soaked into water for three days were measured by oven drying at 105°C for 24 h and the measurements of each kind of gravels were replicated five times. The results showed that *θ_s_* of shale clasts was 0.09±0.02 g g^−1^, while that of pebbles was almost zero. In the sample saturation process, the air bubbles enclosed in the gravels possibly caused the gravel in incomplete saturated and subsequently the underestimation of *θ_s_* in this study.

The clay loam soil was mixed with air-dried shale clasts or pebbles at the ratios of 10%, 15%, 25%, 35%, 45%, 55%, and 65% on a total mass basis in each of the three size classifications (2–3 mm, 3–5 mm, and 5–10 mm). We totally prepared 45 samples including one soil sample, two gravel samples, and 42 soil-gravel mixture samples. The soil-gravel mixtures were packed uniformly by hand into soil containers (98.2 cm^3^ with 5 cm high and 5 cm inner diameter) and the bulk density of clay loam soil without gravel predetermined 1.28 g cm^−3^ for all the soil and soil-gravel mixture samples. Then the soil containers were posited inside the centrifuge rotor chamber (Hitachi-CR21G, Hitachi Ltd. in Japan) under dry conditions (i.e., water content was less than 0.5%). The bulk density of soil-gravel mixtures is presented in Table 2; it was calculated from the measured dry mass and the volume of the samples after finishing centrifuge rolling.

**Table 2:** Packed bulk densities (g cm^−3^) of clay loam soil and gravel mixtures.

Gravel content (%)	Shale clasts			Pebbles		
	2–3 mm	3–5 mm	5–10 mm	2–3 mm	3–5 mm	5–10 mm
0	1.28	1.28	1.28	1.28	1.28	1.28
10	1.31	1.31	1.32	1.35	1.35	1.36
15	1.34	1.35	1.35	1.39	1.39	1.39
25	1.39	1.40	1.40	1.47	1.47	1.48
35	1.45	1.46	1.45	1.56	1.56	1.56
45	1.50	1.50	1.50	1.67	1.66	1.67
55	1.58	1.58	1.58	1.79	1.77	1.78
65	1.63	1.66	1.66	1.93	1.91	1.91
100	1.36	1.39	1.36	1.60	1.61	1.61

The WRCs of soil, gravels, and soil-gravel mixtures were measured separately by the centrifuge method [32], and each treatment had three replications. The tested suction head in the WRCs measurement was in the range of 102 cm to 10200 cm. The Hitachi-CR21G centrifuge was equipped to maintain air temperature constantly at 20°C. For the centrifugation method, soil water desorption was accomplished by applying a high gravity field (centrifugal force) to saturated soil samples. The suction heads were obtained by sequentially increasing the angular velocity or rotation speed of the centrifuge [33]. The rotation speeds and equilibrium times corresponding to the tested pressure heads are shown in Table 3. Water retention properties of the samples near saturation were not considered because the water retention of the artificially packed samples was conditioned by the samples packing and the geometry of soil samples, especially in the low matric suction region [34].

**Table 3:** Rotation speeds and equilibrium times corresponding to the tested pressure heads in the centrifuge method.

Pressure head (cm)	Rotation speed (r s^−1^)	Equilibrium time (min)
102	16.3	26
204	23.1	36
408	32.7	45
612	40	51
816	46.2	55
1020	51.7	58
2040	73.1	68
4080	103.4	77
6120	126.6	83
10200	163.4	90

For those *θ*-*h* data, BC-function and VG-function parameters describing WRCs of soil, gravels, and soil-gravel mixtures were determined by the computer program RETC.FOR [35] using the Marquardt nonlinear least-squares optimization algorithm [36]. BC-function and VG-function are presented as Eq. (1) and Eq. (2):
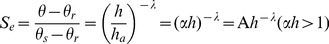
(1)

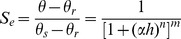
(2)where *S_e_* is the effective degree of saturation, also called reduced water content; *θ_s_* is the saturated water content (cm^3^ cm^−3^); *θ_r_* is the residual water content (cm^3^ cm^−3^); *h* is the matric suction (cm); *α* is the empirical parameter whose inverse equals to *h_a_* and is referred as the air entry value or bubbling pressure (cm^−1^); and *λ* is the pore-size distribution parameter; *n*, *m*, A = *α* ^−*λ*^ are empirical parameters affecting the shape of the retention curve, and *m* = 1–1/*n*.

## Results and Discussion

### Effects of rock fragments on WRCs

**Figure 1:**
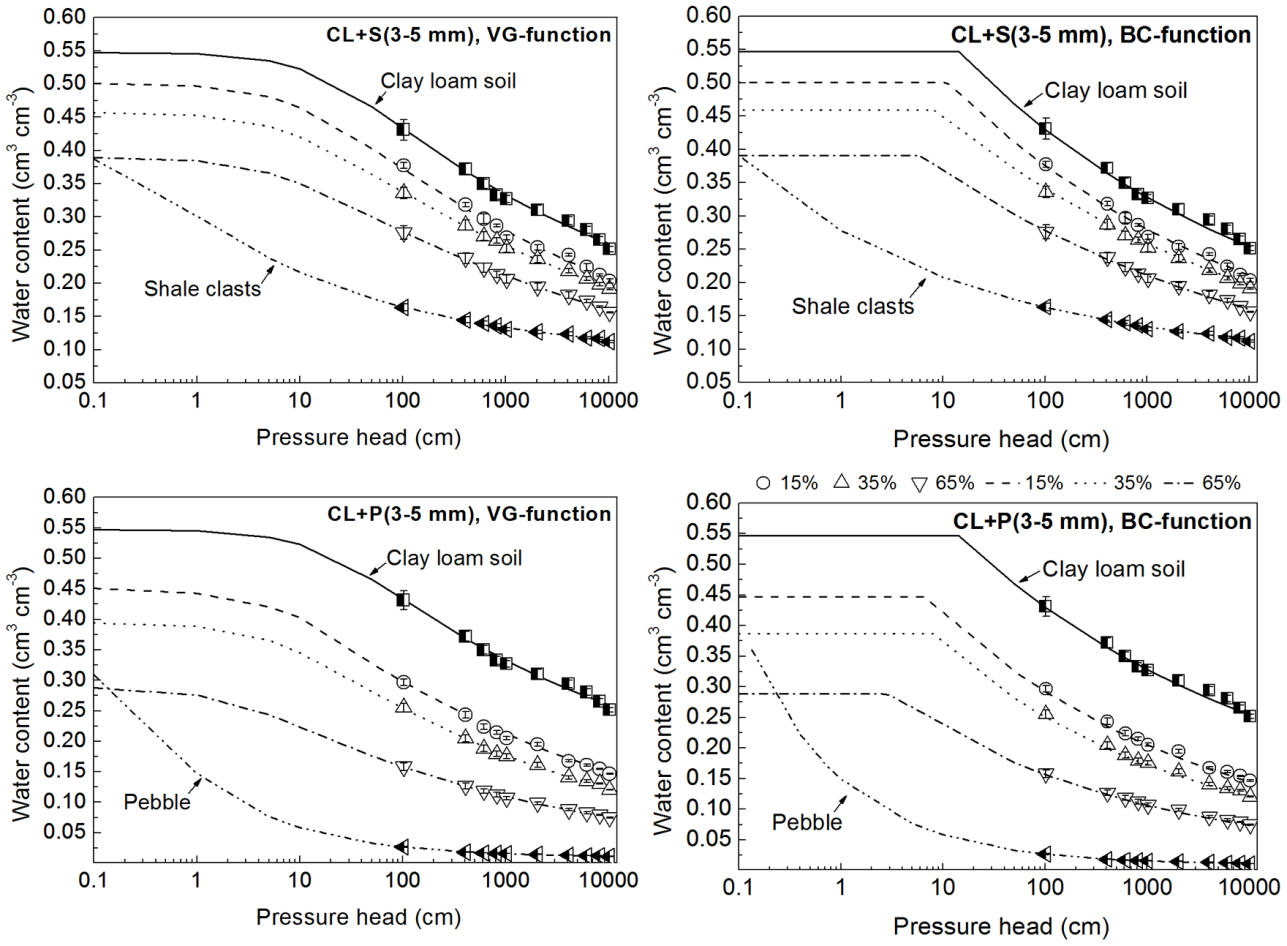
The measured data and fitted water retention curves for soil-gravel mixtures with 3–5 mm gravels. Point: Measured data; Line: Fitted data. CL+S stands for the mixtures of clay loam soil and shale clasts with varied gravel content; CL+P stands for the mixtures of clay loam soil and pebble with varied gravel content. The pressure head in the axe is the absolute value of the actual pressure head which is negative in the measurement.

Soil matric suction (*h*) is a function of water content (*θ*) in an unsaturated soil and WRCs express the relationship between them. The VG-function resolves the coherence problem of WRCs near soil saturation and therefore it has become one of best choices for the analytical model for *θ*(*h*), especially for undisturbed field soil and many fine-texture soils [37], [38]. While BC-function, leads to an air-entry value in the WRC above which soil is assumed to be saturated, has the ability of more accurate description for coarse texture soil with structural deterioration or compaction, especially in the dry end of WRCs [39]. However, in this study, they gave similar fine fitting performance (Fig. 1 and Table 4).

**Table 4:** BC-function parameters describing the water retention characteristics of soils, soil-gravel mixtures, and gravels.

Samples	Gravel content (%)	Gravel size in 2–3 mm					Gravel size in 3–5 mm					Gravel size in 5–10 mm						
		*θ_s_* *	*θ_r_*	1/*α *(cm^−1^)	*λ*	*RMSE *(×10^−3^)	*θ_s_*	*θ_r_*	1/*α *(cm^−1^)	*λ*	*RMSE *(×10^−3^)	*θ_s_*	*θ_r_*	1/*α *(cm^−1^)		*λ*		*RMSE *(×10^−3^)
CL	0	0.55	0.06	14.15	0.14	17.61	-	-	-	-	-	-	-	-	-		-	
CL+S	10	0.52	0.00	12.68	0.11	13.42	0.53	0.00	11.11	0.12	8.37	0.54	0.07	18.62	0.17		12.25	
	15	0.53	0.00	10.16	0.11	14.49	0.50	0.03	10.89	0.14	12.25	0.51	0.04	15.27	0.15		11.83	
	25	0.51	0.00	10.15	0.11	12.25	0.49	0.00	8.58	0.13	12.65	0.48	0.02	13.69	0.14		5.48	
	35	0.48	0.00	8.41	0.11	8.94	0.46	0.00	8.19	0.12	5.48	0.46	0.00	14.65	0.14		13.04	
	45	0.46	0.00	8.25	0.12	10.49	0.43	0.00	8.95	0.12	7.75	0.43	0.00	13.98	0.14		10.49	
	55	0.44	0.00	6.48	0.12	10.00	0.42	0.02	6.51	0.13	7.75	0.41	0.00	9.78	0.13		8.94	
	65	0.44	0.02	5.74	0.13	10.00	0.39	0.00	5.93	0.12	8.37	0.36	0.05	16.74	0.20		9.49	
	100	0.41	0.11	34.71	0.80	7.07	0.41	0.08	0.07	0.19	5.48	0.41	0.00	0.44	0.18		15.49	
CL+P	10	0.48	0.09	21.39	0.27	5.48	0.49	0.09	17.77	0.29	9.49	0.47	0.08	16.77	0.28		7.75	
	15	0.46	0.05	12.55	0.21	13.04	0.45	0.01	6.48	0.16	9.49	0.45	0.02	7.38	0.18		6.32	
	25	0.44	0.02	7.00	0.18	4.47	0.43	0.00	4.37	0.15	8.94	0.43	0.00	4.61	0.16		7.75	
	35	0.44	0.04	6.55	0.21	4.47	0.39	0.03	7.95	0.19	7.07	0.40	0.00	3.84	0.16		6.32	
	45	0.43	0.02	4.07	0.19	7.07	0.37	0.04	6.58	0.22	4.47	0.38	0.03	6.53	0.20		4.47	
	55	0.34	0.04	5.42	0.21	6.32	0.31	0.02	4.78	0.19	4.47	0.33	0.00	3.47	0.15		5.48	
	65	0.33	0.02	4.26	0.21	11.83	0.29	0.01	2.83	0.18	3.16	0.28	0.03	7.76	0.24		5.48	
	100	0.36	0.03	24.21	1.11	4.47	0.36	0.01	0.13	0.46	0.00	0.36	0.00	0.00	0.24		0.00	

* *θ_s_* for soil and soil-gravel samples were the measured value, and *θ_s_* for gravel samples were calculated from density and bulk density. *RMSE*: square root of residual sum of squares values.

The maximum relative standard error (*RSE*m) for the saturated water contents of each sample was lower than 2.5% (n = 3), and the *RSE*m for the unsaturated water contents of them at various pressure heads was lower than 2.2% (n = 3), which showed a good representation for each sample; the measured WRCs data by the centrifuge method had acceptable accuracy for soil-gravel mixtures. While, the measured data of water retention for the soil-gravel samples might be underestimated due to the experimental set up, in which the gravels was possibly not moistured completely under normal air pressure conditions. Parameter sensitivity at one pressure head for WRCs was evaluated by the average value of the total ratios of the relative changes of *θ* to the relative changes of values of one parameter. The results of sensitivity analysis showed that the absolute value of sensitivity for parameter *α* in BC-function increased with soil suction till it reached 1/*α*, and then kept at that maximum value. Correspondingly, that value in VG-function increased with soil suction till it reached the dry end of curve (*h* = 10200 cm, except for the mixture samples containing 55% gravels, it reached at *h = *407 cm). Parameter *n* became most sensitive at *h* = 10200 cm for both BC- and VG-function. The increasing sensitivity of *α* and *n* to BC- and VG-function with the increased suction implied that the water content at *h* = 0 and *h* = 100 cm decided the trend and shape of WRCs in the wet end (h = 0–100 cm), which varied insignificantly when the parameters were changed. Those results indicated that the comparison between WRCs combining extrapolated and measured one for soil-gravel samples was acceptable. It seems that *α* reflected the order of air-entry values from those *α* values (lower, media, upper value at 95% confident limits) for various soil-gravel samples due to its high sensitivity to BC-function at *h* = 1/*α*. One thing should be noted that the measured *θ_s_* and the fitted *θ_r_* in BC- and VG- function were as the fixed value in the analysis of parameter sensitivity.

There were considerable differences between the water retention parameters of soil-gravel mixtures with different gravel contents (Table 4). The air-entry values (1/*α*) of the soil-gravel mixtures decreased gradually with increasing gravel content when it was lower than 55%; the reason may be that the amount of coarse pores increased with gravel content (Although the gravels were packed by hand to increase uniformity, there was still some pores in the soil-gravel mixtures because these gravels possibly overlapped each other and functioned as skeleton). However, when the gravel content reached 65%, especially for the soil-gravel mixtures containing the larger gravels (5–10 mm), the air-entry values increased slightly. It was further noted that the stronger the weathering degree of rock fragments in the soil, the higher the air-entry values, at least in the range of the higher coarse fragment contents for soil-gravel mixtures. Apparently, the shale clasts with smaller sizes had more void characteristics similar to fine soil medium than the pebbles. This observation was confirmed by the fact that the *θ_s_* of clay loam-shale clast mixtures (CL+S) were larger at any gravel content than those of the clay loam-pebble mixtures (CL+P), while this effect was probably aggravated by the side-effect (side conditions in the container) in the experiment. It is reasonable to speculate that the measured *θ_s_* of the soil-gravel mixtures decreased linearly with the increase of rock fragment contents because the majority of water was held by the fine soil of soil-gravel mixtures. The variation of *θ_r_* in the soil-gravel mixtures in this research differed from that reported by Indrawan et al. [40], according to whom the residual water content decreased with increasingly coarse fragments.

The WRCs for fine soil, gravel, and soil-gravel mixtures with different gavel contents (15%, 35%, and 65%) are presented in Fig. 1. This figure only displays the results of soil-gravel mixtures with 3–5 mm gravel because of the similarity among the different size groups. It can be seen that the WRCs of the soil-gravel mixtures are located between the WRCs of the pure soil and those of the rock fragment media. According to the fitting curves of VG-function, the slopes of the WRCs for the soil-gravel mixtures increased with the coarse grain contents at a low suction range (0–100 cm). That suggested an increase in the rate of the soil water volume changes with respect to the matric suction changes (*∂θ/∂h*). However, the trend to increase in slopes of the WRCs for the soil-gravel mixtures became less significant with increasing rock fragment contents at the high suction range (100–1000 cm), and became opposite at a higher suction range (>1000 cm), even lower than the slope of WRCs of the fine soils, especially for the soil-pebble mixtures (see CL+P in Fig. 1). This indicated that a soil containing a high amount of rock fragments at lower water contents releases less water than the others, which might impair plant growing.

### Direct calculation of the saturated water content of soil-gravel mixtures

Knowing the saturated water content is a prerequisite for calculating the effective degree of saturation. At or near saturation, the moisture of the soil-gravel mixtures equals the sum of water amount hold by the fine soil and that by the rock fragments. As a result, the water content of the soil-gravel mixtures could be predicted according to the saturated water content of the fine soil and the rock fragments, respectively, as well as their content in the soil-gravel mixtures. The formula expressing the saturated water content of soil-gravel mixtures is given in Eq. (3):
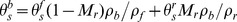
(3)where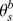, 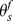, and 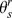 are saturated volumetric water content of the soil-gravel mixture, fine soil, and gravel, respectively (cm^3^ cm^−3^); *M_r_* is the gravimetric gravel content (g g^−1^); *ρ_b_*, *ρ_r_*, and *ρ_f_* is the bulk density of the soil-gravel mixture, fine soil, and gravel, respectively (g cm^−3^); and both superscripts and subscripts refer to bulk sample (soil-gravel mixture) (*b*), fine soil alone (*f*), rock fragments (*r*), and at saturated state (*s*), respectively.

**Figure 2:**
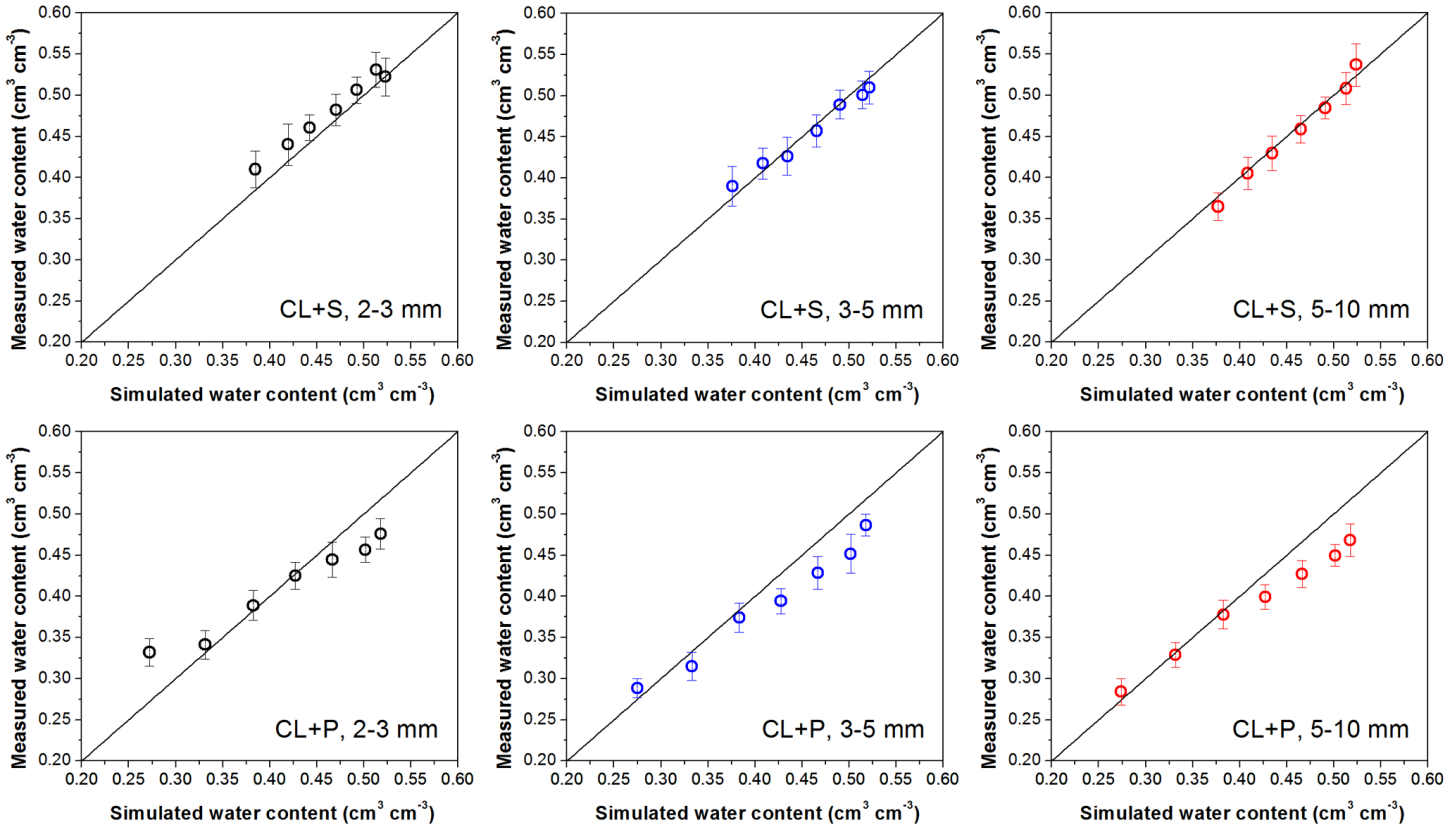
The measured and simulated saturated water contents for soil-gravel mixtures with varied gravel sizes. CL+S stands for the mixtures of clay loam soil and shale clasts; CL+P stands for the mixtures of clay loam soil and pebble.

Reasonably good results were obtained using Eq. (3) to predict the saturated water contents of all the samples, even those containing the weathered rock fragments. The values resulting from Eq. (3) and those actually measured were almost identical for the soil-gravel mixtures with all sizes of gravels (see Fig. 2); a small deviation between the measured water contents and the predicted values for CL+P with relative low gravel content due to measured error was recorded (see CL+P in Fig. 2). Of course, when the 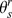 value of the pebbles was assumed to be zero (i.e., Eq. (3) was the same as Bouwer-Rice equation) [20], the predicted results for CL+P with relative low gravel contents could not be improved.

### Revised formulas for water retention processes of soil-gravel mixtures

BC-function with simple power equation, introduces a well-defined air-entry value, which is associated with a largest pore-size through the relation of Young Laplace, assuming complete wettability [41]. In addition, the parameter *α* in BC-function could reflect the variation of air-entry value of varied soil-gravel samples even lack data of wet end of WRC. Due to these reasons, BC-function was selected to be the revised model for soil-gravel mixtures in this study. To examine the moisture content of the unsaturated soil-gravel mixtures, we generated water retention curved surfaces (curve surfaces which reflected the water retention variation of soil-gravel mixtures) by adding one variable to BC-function, and then fitted the empirical parameters of the curved surface based on the measured WRCs data of the soil-gravel mixtures. We found that the effective saturation (*S_e_*) of the soil-gravel mixtures at any soil potential had a reasonable power or linear correlation with the gravimetric gravel content (*M_r_*) or bulk density of the soil-gravel mixtures (*ρ_b_*). Thus *M_r_* and *ρ_b_* were added respectively as further variables of BC-function and the revised water retention functions were established. Eq. (4) and (5) express power relations:
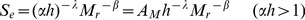
(4)

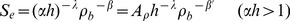
(5)where *A_M_*, *A_ρ_*, *β*, *β'*, and *λ* are empirical parameters. Eq. (6) and (7) express power-linear relations.



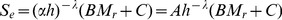
(6)

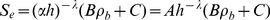
(7)When Eq. (6) and Eq. (7) were simplified to decrease the number of the parameters, the water retention curved surfaces were expressed as Eq. (8) and Eq. (9):



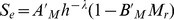
(8)

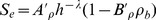
(9)where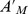, 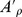, 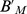, and 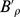
are empirical parameters.

The water content of the soil-gravel mixtures can be calculated according to the revised water retention functions and the definition of *S_e_*. By taking both Eq. (5) and Eq. (8) as examples, the water content of the soil-gravel mixtures can be calculated by the formulas listed as follows.
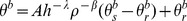
(10)

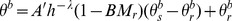
(11)


The saturated water content of soil-gravel mixtures (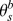) in Eq. (10) and Eq. (11) could be obtained by direct measurement in the laboratory, while it can be calculated indirectly using Eq. (3) above if pertinent detailed information is known, such as gravel content, sample bulk density, gravel density, saturated water content of fine soil, and water content of gravels.

### Parameter determination for the proposed revised formulas through nonlinear regression for the soil-gravel mixtures

Two variables (*Mr* and *ρb*) which presented a linear or power relation with *Se* were added to BC-function. In order to test if those revised water retention functions were practical and applicable for obtaining the WRCs parameters of the soil-gravel mixtures, we used the measured data to fit the revised formulas and obtain the parameters of curved surfaces by a nonlinear regression.

#### Parameter determination for separate classes of the gravel sizes

**Figure 3:**
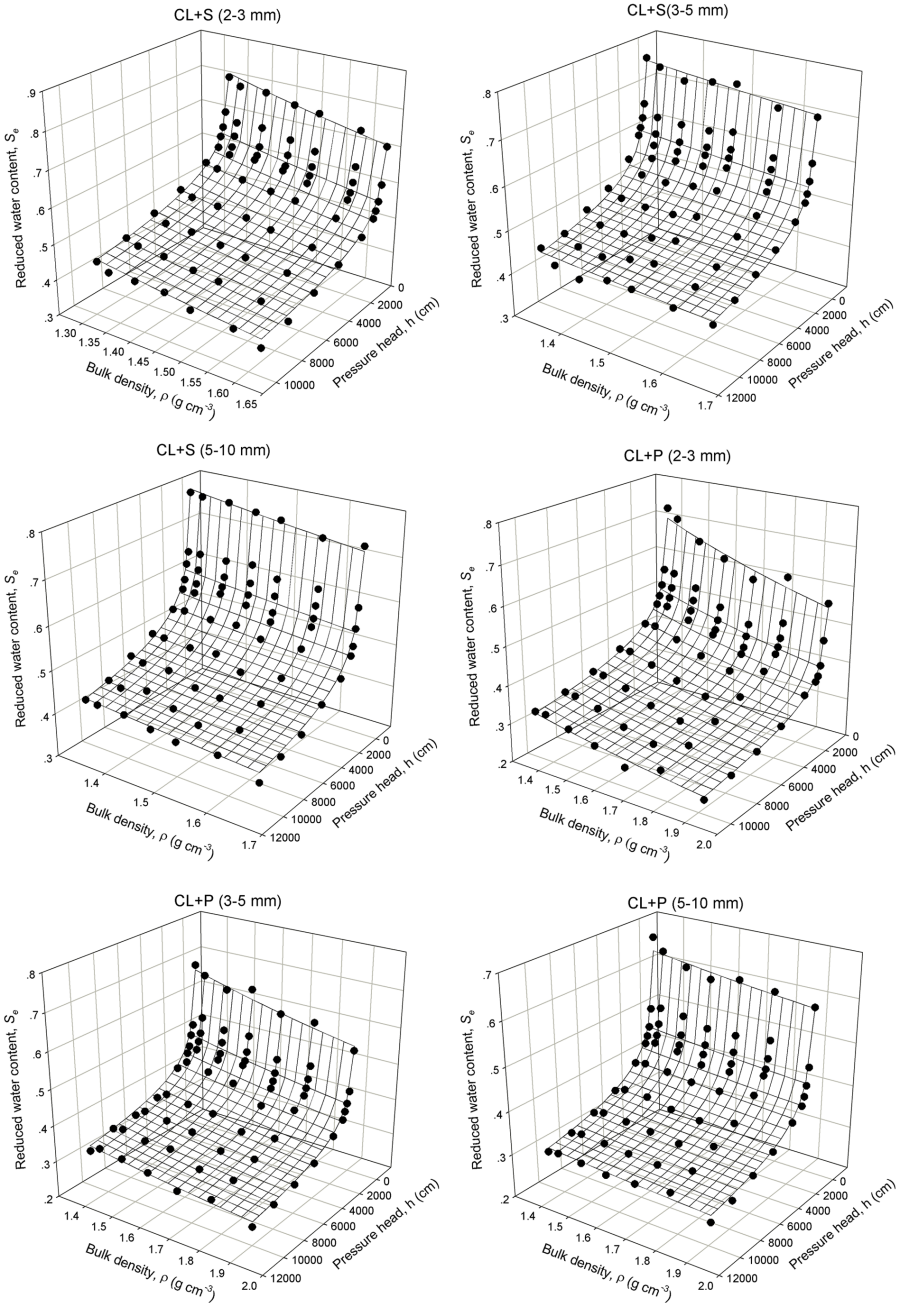
The measured points and the regression surfaces of power function with variable of samples bulk density for soil-gravel mixtures (•: Measured point; mesh: Regression surface). The pressure head in the axe is the absolute value of the actual pressure head which is negative in the measurement.

The fitting results are given in Table 5 and Table 6. It is shown that the values of coefficient of determination (*R*2), estimated standard error (*SEE*), and square root of the residual sum of squares (*RSME*), which all reflect the nonlinear regression matching level, indicate that the fitting results were in considerable agreement for both the power function and the linear-power function curved surfaces with either selected variable (*M_r_* and *ρb*). Though the values of *R*2 and *SEE* for the different curved surfaces of the various soil-gravel mixtures were similar (Table 5 and Table 6), the difference of *Norm* indicated that the power function with the variable of sample bulk density (Eq. (5)) and linear-power function with the variable of gravimetric gravel content gave a better fitting (Eq. (8)). The regressed curved surfaces of power function with the variable “bulk density” taken as an example here are drawn in Fig. 3 showing the regression results based on the measured data. The two different types of revised formulas, with the variables bulk density or gravel content, provided options for spatial heterogeneous research of regional soil hydrology according to the types of the practical measured data. The above formulas and the empirical parameters listed in Table 5 and Table 6 concur to simplify the determination of variability in water retention properties for soil-gravel mixtures with highly variable gravel content in soilscape.

**Table 5:** Regression empirical parameters for curved surface functions with *M_r_* obtained by fitting all the measured water retention curves.

Samples	*S_e_* = *A_M_h* ^−*λ*^ *M_r_* ^−*β*^						*S_e_* = *A_M_'h* ^−*λ*^(*B_M_'M_r_*+1)					
	*A_M_*	*λ*	*β*	*R*2	*SEE*	*REMS*	*A_M_'*	*λ*	*B_M_'*	*R*2	*SEE*	*REMS*
CL+S (2–3)	1.18	0.12	0.07	0.97	0.01	0.13	1.39	0.12	0.23	0.98	0.01	0.10
CL+S (3–5)	1.25	0.12	0.03	0.98	0.01	0.10	1.33	0.12	0.09	0.98	0.01	0.10
CL+S (5–10)	1.36	0.14	0.03	0.97	0.01	0.12	1.48	0.14	0.13	0.98	0.01	0.11
CL+S (2–10)	1.26	0.12	0.04	0.96	0.02	0.29	1.40	0.12	0.15	0.97	0.02	0.28
CL+P (2–3)	1.12	0.16	0.13	0.98	0.02	0.12	1.52	0.16	0.39	0.98	0.02	0.12
CL+P (3–5)	1.15	0.16	0.08	0.97	0.02	0.15	1.41	0.16	0.28	0.98	0.01	0.11
CL+P (5–10)	1.24	0.16	0.04	0.97	0.01	0.11	1.36	0.16	0.14	0.98	0.01	0.11
CL+P (2–10)	1.17	0.16	0.08	0.96	0.02	0.27	1.43	0.16	0.27	0.97	0.02	0.25

*R*2, *SEE*, and *REMS* stand for coefficient of determination, estimated standard error, and square root of the residual sum of squares, respectively.

**Table 6:** Regression empirical parameters for curved surface functions with *ρ* obtained by fitting all the measured water retention curves.

Samples	*S_e_* = *A_ρ_h* ^−*λ*^ *ρ* ^−*β'*^						*S_e_* = *A_ρ_'h* ^−*λ*^(*B_ρ_'ρ*+1)					
	*A_ρ_*	*λ*	*β'*	*R*2	*SEE*	*REMS*	*A_ρ_'*	*λ*	*B_ρ_'*	*R*2	*SEE*	*REMS*
CL+S (2–3)	1.61	0.12	0.62	0.98	0.01	0.10	2.07	0.12	0.26	0.98	0.01	0.10
CL+S (3–5)	1.41	0.12	0.13	0.98	0.01	0.08	1.59	0.12	0.08	0.98	0.01	0.10
CL+S (5–10)	1.60	0.14	0.32	0.98	0.01	0.10	1.87	0.14	0.17	0.97	0.01	0.12
CL+S (2–10)	1.54	0.12	0.40	0.97	0.02	0.26	1.85	0.12	0.19	0.97	0.02	0.27
CL+P (2–3)	1.81	0.16	0.71	0.98	0.02	0.12	2.20	0.16	0.25	0.98	0.02	0.14
CL+P (3–5)	1.59	0.16	0.50	0.98	0.01	0.11	1.90	0.16	0.21	0.98	0.01	0.10
CL+P (5–10)	1.45	0.16	0.23	0.98	0.01	0.10	1.60	0.16	0.12	0.98	0.01	0.11
CL+P (2–10)	1.61	0.16	0.48	0.97	0.02	0.24	1.91	0.16	0.20	0.97	0.02	0.24

*R*2, *SEE*, and *REMS* stand for coefficient of determination, estimated standard error, and square root of the residual sum of squares, respectively.

#### Model effectiveness and parameter determination for soil-gravel mixtures with distribution of the three classes of gravel sizes

**Figure 4:**
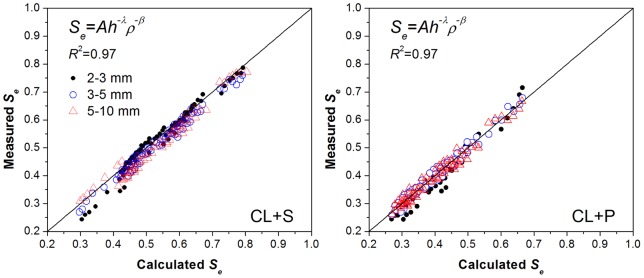
The measured and fitted data calculated by the trade-off parameters of power surface model with the variable of samples bulk density without considering gravel size change.

**Figure 5:**
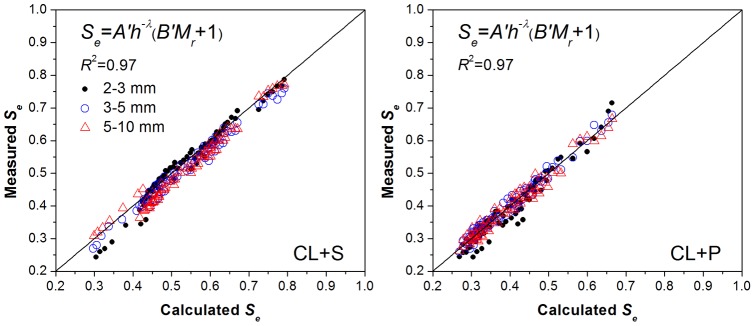
The measured and fitted data calculated by the trade-off parameters of linear-power surface model with the variable of gravel mass content without considering gravel size change.

Table 5 and Table 6 show that the empirical parameters for various soil rock fragments mixtures vary with gravel type and size. The shape parameter *λ* for soil mixtures containing the same type of gravels with three size groups showed little difference, while by comparing the *λ* values between the two kinds of gravels it was observed that the stronger the weathering degree of the rock fragments in the soil-gravel mixtures, the smaller the *λ* values. The shape parameter *β* or *B* in the power function or linear-power function, respectively, was decreasing with increasing gravel sizes, while the differences in *β* or *B* between different size groups were not significant. Therefore, it seemed reasonable to consider obtaining the “trade-off” and appropriate parameters for the curved surfaces to account for the water retention properties of soil-gravel mixture with a large range of gravel size (2–10 mm). The fitting parameters, obtained by fitting the measured data of soil-gravel mixtures within all size groups (2–3 mm, 3–5 mm, and 5–10 mm) for the same kind of gravels, are given in Table 5 and Table 6. The fitting results of the soil-gravel mixtures containing the large range of the gravel sizes from 2 to 10 mm were also rather satisfactory since the *R*2 values of the two typical soil-gravel mixtures were both 0.97. The match effect between measured and calculated data is presented respectively in Fig. 4 and Fig. 5 using the trade-off parameters according to Eq. (5) and Eq. (8). Both Fig. 4 and Fig. 5 show that there was a good match between the measured and simulated data. In addition, the two kinds of the revised WRCs formulas (Eq. (5) and Eq. (8)) had almost the same fitting level. The well fitted results for the soil-gravel mixtures with the gravel sizes of 2–10 mm lead to conclude that the effect of gravel sizes was much lower than that of the gravel contents and types. This conclusion gives support to the effectiveness of revised models for large scale field investigation of the soil-gravel mixtures with highly variable gravel size.

#### Parameter determination with two data sets of representative gravel contents

**Figure 6:**
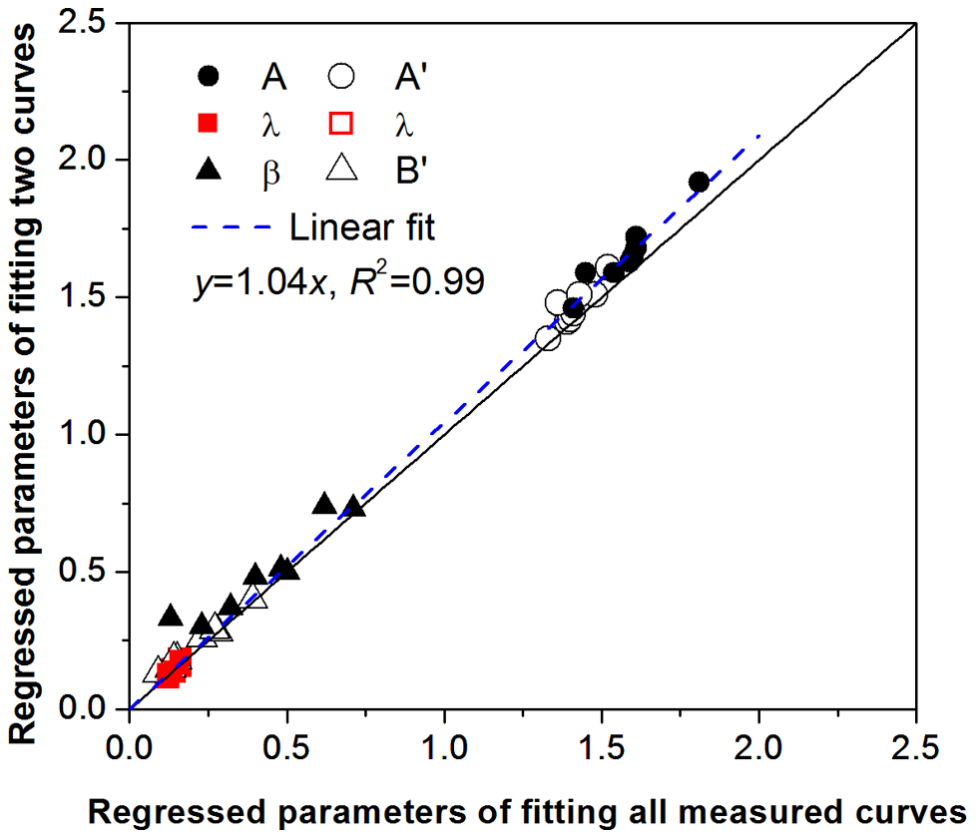
Comparison between the regressed parameters by fitting two and all measured water retention curves of soil-gravel mixtures.

As shown above, the water characteristic properties of the soil-gravel mixtures can be well determined based on the seven WRCs data sets corresponding to the seven different gravel mass contents (10%, 15%, 25%, 35%, 45%, 55%, and 65%) by fitting the regression surfaces, but the WRCs measurement work for seven gravel mass contents might be time-consuming. It was explored consequently if two measured water retention curves of soil mixtures with minimum and maximum gravel contents (10% and 65%) could represent well all the measured data within the whole range of gravel contents (10%–65%) in determining the parameter of revised water retention model. To examine that, the measured water retention curves of the soil-gravel mixtures with 10% and 65% gravel contents were used to fit Eq. (5) and Eq. (8), respectively, by the nonlinear regression. The regression results are reported in Table 7 showing that high *R*2 values were achieved. Importantly, the values of the empirical fitting parameters from the two measured curves were rather close to those from the whole measured curves, which indicated that the curved surface obtained based on the two measured curves were acceptable. The comparison between fitting parameters calculated from the two data sets and those from the seven data sets is presented in Fig. 6. The parameters of *A* and *A’* for Eq. (5) and Eq. (8) obtained from fitting the two curves were slightly larger than those from fitting the whole measured curves. However, the small deviation did not bring about a considerable difference between the two groups of the curve surface parameters. Therefore the method of determining the parameters of the revised water retention model based on the measured data from two representative gravel contents can be recommended, probably being more practical for the mixtures with embedding gravels in fine soil and for poorly sorted sediments with small size gravels in regional or large scale field investigation.

**Table 7:** Regression empirical parameters obtained by fitting two measured water retention curves of soil mixtures with 10% and 65% gravel content.

Samples	*S_e_* = *Ah* ^−*λ*^ *ρ* ^−*β*^						*S_e_* = *A'h* ^−*λ*^(*B'M_r_*+1)					
	*A*	*λ*	*β*	*R*2	*SEE*	*REMS*	*A'*	*Λ*	*B'*	*R*2	*SEE*	*REMS*
CL+S (2–3)	1.68	0.12	0.74	0.98	0.01	0.04	1.41	0.12	0.26	0.98	0.01	0.04
CL+S (3–5)	1.46	0.12	0.33	0.98	0.01	0.04	1.35	0.12	0.13	0.99	0.01	0.03
CL+S (5–10)	1.65	0.14	0.37	0.98	0.01	0.05	1.51	0.14	0.15	0.98	0.01	0.05
CL+S (2–10)	1.59	0.13	0.48	0.96	0.02	0.18	1.42	0.13	0.18	0.96	0.02	0.18
CL+P (2–3)	1.92	0.17	0.73	0.98	0.01	0.05	1.61	0.17	0.40	0.98	0.01	0.05
CL+P (3–5)	1.63	0.16	0.50	0.98	0.01	0.04	1.44	0.16	0.28	0.98	0.01	0.04
CL+P (5–10)	1.59	0.18	0.30	0.98	0.01	0.10	1.48	0.18	0.18	0.98	0.01	0.04
CL+P (2–10)	1.72	0.17	0.51	0.97	0.02	0.16	1.51	0.17	0.29	0.96	0.02	0.17

*R*2, *SEE*, and *REMS* stand for coefficient of determination, estimated standard error, and square root of the residual sum of squares, respectively.

## Conclusion

Based on this investigation it can be seen that the saturated water contents of the soil-gravel mixtures can be directly calculated from the amount and saturated water contents of both fine soil and rock fragments. The experimental results achieved from the different samples containing typical rock fragments with various sizes (2–10 mm) confirmed that the given equation was applicable for computing the water content of the soil-gravel mixtures. Revised formulas for water retention processes of the soil-gravel mixtures were proposed. Revised water retention models were developed by adding one variable (gravimetric gravel content or bulk density of soil-gravel mixtures that reflect the change in rock fragment contents) which had linear or power relation with the effective saturation of soil-gravel mixture to BC-function forming curved surfaces. Furthermore, the laboratory results indicated that the revised water retention models calibrated either by using the experimental data from the soil-gravel mixtures with a small range of gravel sizes or with a large range of gravel sizes, would be acceptable. However, determination and application of the regression surface model based on the data sets from a few typical different gravel sizes with two representative gravel contents would be more practical. Finally, it seems that the revised water retention models, the procedure of calculation parameters and the specific parameter values of the curved surfaces obtained in this study may be applied to different kinds of soil-gravel mixtures, because the whole analyses in this study were based on the measured water retention in soil-gravel mixtures containing two different types of rock fragments. However, their applicability needs to be validated through the water retention data from soils containing different rock fragments.
